# Culture substrate‐associated YAP inactivation underlies chondrogenic differentiation of human induced pluripotent stem cells

**DOI:** 10.1002/sctm.20-0058

**Published:** 2020-08-21

**Authors:** Akihiro Yamashita, Hiroyuki Yoshitomi, Shunsuke Kihara, Junya Toguchida, Noriyuki Tsumaki

**Affiliations:** ^1^ Cell Induction and Regulation Field, Department of Clinical Application, Center for iPS Cell Research and Application (CiRA) Kyoto University Kyoto Japan; ^2^ Department of Cell Growth and Differentiation, Center for iPS Cell Research and Application Kyoto University (CiRA) Kyoto Japan; ^3^ Department of Regeneration Science and Engineering, Institute for Frontier Life and Medical Sciences Kyoto University Kyoto Japan; ^4^ Department of Fundamental Cell Technology, Center for iPS Cell Research and Application (CiRA) Kyoto University Kyoto Japan

**Keywords:** cartilage, chondrocyte, chondrogenesis, differentiation, iPSCs, pluripotent stem cells, stem cell culture

## Abstract

Human induced pluripotent stem cells (hiPSCs) are a promising cell source for the creation of cartilage to treat articular cartilage damage. The molecular mechanisms that translate culture conditions to the chondrogenic differentiation of hiPSCs remain to be analyzed. To analyze the effects of culture substrates, we chondrogenically differentiated hiPSCs on Matrigel or laminin 511‐E8 while holding the composition of the chondrogenic medium constant. Cartilage was formed from hiPSCs on Matrigel, but not on laminin 511‐E8. On Matrigel, the hiPSCs were round and yes‐associated protein (YAP) was inactive. In contrast, on laminin 511‐E8, the hiPSCs were flat and YAP was active. Treating the laminin 511‐E8 hiPSCs in a bioreactor caused cell aggregates, in which the cells were round and YAP was inactive. Subsequent culture of the aggregates in chondrogenic medium resulted in cartilage formation. Transient knockdown of YAP in hiPSCs around the start of chondrogenic differentiation successfully formed cartilage on laminin 511‐E8, suggesting that the activation of YAP is responsible for the failure of cartilage formation from hiPSCs on laminin 511‐E8. Consistently, the addition of YAP inhibitors to laminin 511‐E8 hiPSCs caused partial cartilage formation. This study contributes to identifying the molecules that mediate the effects of culture substrates on the chondrogenic differentiation of hiPSCs as well as to developing clinically applicable chondrogenic differentiation methods.

## Graphical abstract

The chondrogenic differentiation of human induced pluripotent stem cells (hiPSCs) was highly efficient with the use of Matrigel as the culture substrate, but was compromised with the use of laminin fragments. Inactivation of YAP by cytosolic transfer made the cell morphology round and improved the chondrogenic differentiation of hiPSCs on the laminin fragments. The use of bioreactors for clinical application reproduced the round cell shape and improved chondrogenic differentiation.

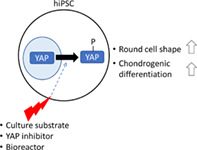

**Significance statement**

Human induced pluripotent stem cells (hiPSCs) are a promising cell source for the creation of cartilage to treat articular cartilage damage. However, it has not been well studied how culture substrates affect the chondrogenic differentiation of hiPSCs. This study revealed that the use of laminin fragments as the culture substrate caused poor chondrogenesis from hiPSCs and that inactivation of yes‐associated protein made the cell morphology round and improved the cartilage formation. This study contributes to identifying the molecules that mediate the effects of culture substrates on the chondrogenic differentiation of hiPSCs as well as to developing clinically applicable chondrogenic differentiation methods.

## 1 INTRODUCTION

Cartilage consists of chondrocytes embedded in cartilage extracellular matrix (ECM). ECM is composed of three‐dimensional networks of types II and XI collagen fibrils, which provide scaffolding for proteoglycan, which is composed of aggrecan and glycosaminoglycan. Articular cartilage has limited capacity for repair, and its damage due to trauma can lead to debilitating conditions such as osteoarthritis. Although autologous chondrocyte implantation^1^ and allogeneic cartilage implantation^2^ have shown good clinical results, they are respectively limited by a small number of chondrocytes and scarcity of donors. On the other hand, because induced pluripotent stem cells (iPSCs) can be almost infinitely expanded without compromising their differentiation capacity,^3^ they are a promising cell source for cartilage to treat articular cartilage damage.

Methods for the chondrogenic differentiation of iPSCs have been extensively studied^4^ and are classified into four categories^5^, ^6^, ^7^: (a) the coculture of iPSCs with primary chondrocytes; (b) the preparation of EBs from iPSCs, followed by the differentiation of mesodermal cells in the EBs into chondrocytes; (c) the induction of mesenchymal stem cell‐like cells from iPSCs, followed by their differentiation into chondrocytes; and (d) the differentiation of iPSCs toward chondrocytes by treating the cells when cultured on dishes coated with culture substrates with chondrogenic growth factors. Among the four categories, recent studies suggest the use of chondrogenic growth factors and culture substrates can improve the reproducibility and efficiency of chondrogenic differentiation.^8^, ^9^, ^10^, ^11^, ^12^ Although it is known that certain combinations of chondrogenic growth factors and culture substrates produce cartilage more efficiently than others, which intracellular molecules mediate the efficient chondrogenic differentiation of human (h)iPSCs remain elusive.

The Hippo (Hpo) pathway consisting of mammalian Ste2‐like kinases (MST1/2, Hpo orthologs) and large tumor suppressor kinase 1/2 (LATS1/2) and its nuclear transducer, yes‐associated protein (YAP) and transcriptional coactivator with PDZ binding motif (TAZ), regulate cell proliferation and organ growth.^13^ Activation of the Hippo pathway results in phosphorylation of YAP/TAZ by LATS1/2, sequestering YAP/TAZ in the cytoplasm and inactivating YAP/TAZ. The activation of YAP has been shown to help maintain the pluripotent and undifferentiated properties of ESCs and iPSCs.^14^, ^15^

The use of defined substances instead of undefined biological substances is one approach for using human induced pluripotent stem cell (hiPSC)‐derived cells/tissues including cartilage clinically.^16^ For example, laminin fragments^17^, ^18^ were developed to replace Matrigel, which is derived from mouse Engelbreth‐Holm‐Swarm tumors and contains many unknown components. In the present study, we found that cartilage was produced less efficiently from hiPSCs when using laminin fragment 511‐E8 instead of Matrigel. We also found that cell morphology on Matrigel was rounder than that on laminin 511‐E8. Because YAP was reported to correlate with the culture substrates and cell morphology of some cell types such as mesenchymal stem cells,^19^, ^20^ we examined its activity in these two conditions. By performing knockdown experiments, we identified the inactivation of YAP as responsible for efficient cartilage formation from hiPSCs.

## 2 MATERIALS AND METHODS

### 2.1 Maintenance of hiPSCs

Two feeder‐free hiPSC lines, QHJI^21^ and 604B1,^21^ were routinely maintained on laminin 511‐E8 (Nippi)‐coated dishes in ESC medium (StemFit AK02N medium (Ajinomoto) with 50 units/mL penicillin and 50 mg/mL streptomycin (Thermo)). hiPSCs were passaged every 7 days by dissociating with Accutase (Nakalai). 10 μM Y‐27632 (Nakalai) was added during the first 3 days after every passage. The medium was changed every 2 to 3 days.

Laminin 511‐E8‐coated dishes were prepared by incubating 3.5 cm culture dishes (Iwaki) with laminin 511‐E8 (0.25 μg/cm^2^) in phosphate buffered saline (PBS) for more than 1 hour at 37°C. Matrigel‐coated dishes were prepared by incubating the dishes with Matrigel (1:100 dilution) (Corning) in DMEM/F12 overnight at 4°C.

QHIJI was used for all the experiments except for those in Figure 6D‐F, in which 604B1 was used.

### 2.2 Chondrogenic differentiation of hiPSCs using laminin 511‐E8‐ or Matrigel‐coated dishes

In the condition in which Matrigel‐coated dishes were used, culture substrates were changed from laminin 511‐E8 to Matrigel (day −7). Chondrogenic differentiation was performed following a previously reported method with some modification.^12^ Seven days later (day 0), the medium was changed from ESC medium to chondrogenic medium #1 (DMEM [Sigma] with 1% Insulin‐Transferrin‐Selenium (ITS) [Thermo], 1% fetal bovine serum (FBS) [Thermo], 1 × 10^−4^ M nonessential amino acids [Thermo], 1 mM Na pyruvate [Thermo], 50 units penicillin and 50 mg/mL streptomycin, 50 μg/mL Ascorbic acid [Nakalai], 10 ng/mL BMP2 [Peprotech], 10 ng/mL TGFβ [Peprotech] and 10 ng/mL GDF5 [Biovision]) to differentiate the hiPSCs to chondrocytes. Fourteen days later (day 14), the cells were physically separated from the bottom of the dishes to form cartilaginous particles, which were then transferred to a suspension culture in 3.5‐cm petri dishes (Corning). After a further 14 days of culture (day 28), particles were harvested and subjected to histological and mRNA expression analyses.

### 2.3 Chondrogenic differentiation of hiPSCs using bioreactors

hiPSCs (1.0 × 10^6^ to 2.0 × 10^6^ cells) in ESC medium containing 10 μM Y‐27632 were put into a 100 mL vessel bioreactor (ABLE) and cultured following the manufacturer's instructions.^22^ After 4 days of cultivation in the bioreactor, the hiPSCs formed aggregates (day 0). The hiPSC aggregates were transferred into ten 10‐cm suspension culture dishes (SUMITOMO BEKLITE) and cultured in chondrogenic medium #2, which is identical to chondrogenic medium #1 except that the FBS concentration was 0.2%. After transferring to suspension culture dishes, the aggregates initially attached loosely to the bottom of the dishes. Fourteen days later (day 14), the loosely attached aggregates were physically separated from the bottom and subjected to suspension culture to form cartilaginous particles. After a further 14 days of culture (day 28), the particles were harvested and subjected to analyses.

### 2.4 YAP knockdown by short hairpin RNAs and siRNAs

A doxycycline‐inducible YAP short hairpin RNA (shRNA) knockdown construct in piggyBac vector was prepared. BamHI and EcoRI sites were introduced into the cloning site of pENTR4‐H1tetOx1 (Riken RDB04395) to create pENTR4‐H1tetOx1BE. The Blasticidin‐resistance gene sequence in CS‐Rfa‐ETBsd (Riken RDB07917) was replaced with the puromycin‐resistance gene sequence to prepare CS‐Rfa‐ETPII. pENTR4‐H1tetOx1BE was recombined with CS‐Rfa‐ETPII by LR reaction to prepare CS‐H1tetOxBE‐ETPII. H1tetOx‐ETP dissected from CS‐H1tetOxBE‐ETPII, PiggyBac transposon ITR sequences retrieved from KW111 (addgene #80475), and the shYAP target sequence (GCCACCAAGCTAGATAAAGAA) were linked to prepare the pPB‐H1TetOx1‐shYAP vector. 1 × 10^6^ hiPSCs suspended in a single‐cell manner in Opti‐MEM medium (Thermo) were transfected with the pPB‐H1TetOx1‐shYAP vector and the transposase expression vector (PBaseII, P16‐25) by using NEPA21 (Nepa Gene Co., Ltd) electroporation at 125 V and a poring pulse length of 5 ms. The cells were subjected to selection with puromycin, and an hiPSC line bearing the vector sequence was established.

siRNAs that target two different sites in the *YAP* RNA sequence (J‐012200‐05 and J‐012200‐06) and nontargeting control siRNA (D‐001810‐01) were purchased from Dharmacon. 2.0 μg siRNA were transfected into 1 × 10^6^ hiPSCs by using NEPA21 following the manufacturer's instructions.

### 2.5 Confocal microscope analysis

Cells were cultured on glass bottom dishes (Iwaki) and fixed with 4% paraformaldehyde for 10 minutes. After washing with Tris‐buffered saline, specimens were permeabilized with 0.1% Triton X‐100 for 10 minutes. After blocking with 10% horse serum/PBS for 30 minutes, the specimens were incubated first with primary antibodies specific to YAP1 (1:2000, Novus, NB110‐58358) overnight at 4°C and then Alexa Fluor conjugated secondary antibody (1:1000, Thermo Fisher Scientific), CytoPainter Phalloidin‐iFlour 555 for F‐actin (1:1000, Abcam 176 756) and 4',6‐diamidino‐2‐phenylindole (DAPI) (1:1000, Dojindo) for 1 hour. Cytoplasmic membranes were stained with Cellbrite (1:200, Biotume 30 023) and Hoechst 33342 (1:1000, WAKO) in live adherent cells according to the manufacturer's instructions. Stained samples were observed using Nikon A1R MP (Nikon).

### 2.6 Real‐time reverse transcription‐polymerase chain reaction (RT‐PCR)

hiPSC‐derived particles were homogenized using a multibeads shocker (Yasui kikai). Total RNA was isolated using the Isogen (Nippon Gene) and RNeasy Mini Kit (Qiagen) with the RNase‐Free DNase Set (Qiagen) according to the manufacturer's instructions. A total of 500 ng RNA was used as a template for the cDNA synthesis using ReverTra Ace (TOYOBO). Real‐time PCR was performed in a Step One system (ABI) using a KAPA PROBE FAST qPCR kit Master Mix ABI prism (KAPA BIOSYSTEMS). The primers used were *GAPDH*: Hs03929097_g1, *OCT4* (POU5F1): Hs01654807_s1, *NANOG*: Hs04260366_g1, *LIN28A*: Hs00702808_s1, *SOX9*: Hs01001343_g1, *COL2A1*: Hs00264051_m1, and *ACAN*: Hs00153936_m1 (ABI). The amplified products were used to derive standard curves for quantitative real‐time PCR. The mRNA expression levels were normalized to the level of the *GAPDH* expression.

### 2.7 Western blotting

Cells were lysed in RIPA buffer (Thermo) and subjected to sodium dodecyl sulfate (SDS) page. The separated proteins were then electroblotted and immunostained with rabbit anti‐YAP antibody (1:1000, Novus, NB110‐58358), rabbit anti‐phospho‐YAP antibody (1:500, Cell Signaling, #13008), and rabbit anti‐β‐actin antibody (1:2500, Cell Signaling, #4967) overnight at 4°C. After washing, the immunostained membranes were incubated with a 1:5000 dilution of horseradish peroxidase‐conjugated anti‐rabbit IgG antibodies (Thermo) for 1 hour. The SuperSignal West Dura Extenden Duration Substrate (Thermo) and LAS4000 (GE Healthcare) were used for chemiluminescent immunodetection.

### 2.8 Histology

hiPSC‐derived cartilaginous particles were fixed with 4% paraformaldehyde, processed and embedded in paraffin. Semiserial sections were stained with hematoxylin‐eosin and safranin O‐fast green‐iron hematoxylin. The area of the Safranin O‐positive region and the total area of the particle were measured using Image J.

### 2.9 Preparation of YAP inhibitors

Stock solutions of Dasatinib (BioVison #1586) and Vertephorfin (Sigma SML0534) were prepared by dissolving the drugs in DMSO at concentrations of 0.1, 1, and 10 μM. Aliquots of stock solutions were added to the culture medium. The final concentrations were 0.1, 1, and 10 nM. As a control, an equal amount of dimethyl sulfoxide (DMSO) was added to the medium (vehicle).

### 2.10 Statistical analysis

The data are shown as means and SDs. We used Student's *t* test. *P* values <.05 were considered statistically significant.

## 3 RESULTS

### 3.1 Association between Matrigel substrate, YAP inactivation, and cartilage formation from hiPSCs

**FIGURE 1:**
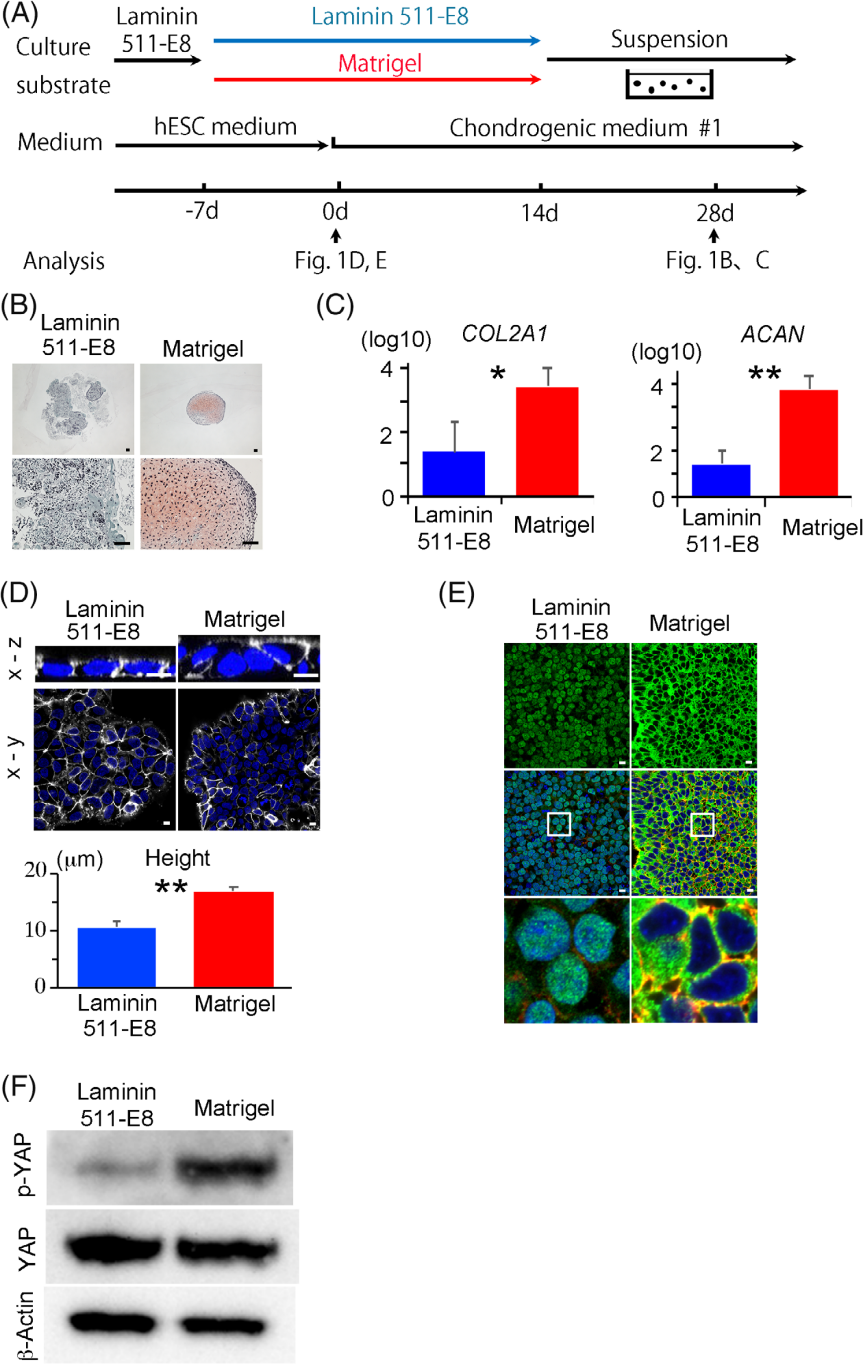
hiPSCs cultured and differentiated into cartilage in laminin 511E8‐ or Matrigel‐ coated dishes. A, Scheme for the chondrogenic differentiation of hiPSCs using either laminin 511E8‐ or Matrigel‐coated dishes. B, Histological sections of particles on day 28 were stained with Safranin‐O. Scale bars = 50 μm. One image is representative of three particles. C, Real‐time reverse transcription‐polymerase chain reaction (RT‐PCR) measurement of *COL2A1* and *AGGRECAN* (*ACAN*) expression in particles on day 28 (N = 3 independent experiments). Student's *t* test. *P < .05. **P < .01. D‐F, hiPSCs were cultured on laminin 511‐E8 or Matrigel for 7 days and subjected to analysis. D, *Top*, Cell membrane staining using Cellbrite. Snapshots of the 3D reconstitution display from a z‐series of confocal images are shown. The upper and bottom images are the x‐z and x‐y views, respectively. Cell membrane (white) and Hoechst (blue). Scale bars = 10 μm. *Bottom*, Height of the hiPSC colonies (N = 6 microscope fields in one dish). The data are representative of two independent experiments. Student's *t* test. **P < .01. E, Immunostaining for YAP in confocal imaging. *Top*, Anti‐YAP antibody (Green); *Middle*, overlay with 4',6‐diamidino‐2‐phenylindole (DAPI) (Blue) and anti F‐actin (Red); *Bottom*, High magnification of the squares in *Middle*. Scale bars = 10 μm. The data are representative of two independent experiments. F, Western blotting for phosphorylated YAP (pYAP), total YAP and β‐actin. The data are representative of three independent experiments. hiPSCs, human induced pluripotent stem cells; YAP, yes‐associated protein

hiPSCs were maintained on laminin 511‐E8 (culture substrate) in ESC medium. To examine the difference in chondrogenic differentiation between laminin 511‐E8 and Matrigel, we replated the QHJI hiPSCs onto either laminin 511‐E8‐ or Matrigel‐coated dishes and cultured them in ESC medium for 7 days. Then the medium was changed to chondrogenic medium #1. Fourteen days later, we detached the cells from the dishes and transferred them into suspension culture for another 14 days in a petri dish to form cartilaginous particles in chondrogenic medium #1 (Figure 1A). The particles produced from cells cultured on laminin 511‐E8 were not cartilaginous, but those produced from cells cultured on Matrigel were, as indicated by histological sections stained with safranin O, which stains glycosaminoglycan in cartilage ECM (Figure 1B). Consistently, the mRNA expression levels of the chondrogenic markers *COL2A1* and *AGGRECAN (ACAN)* were higher in particles generated from the Matrigel group (Figure 1C).

**FIGURE 2:**
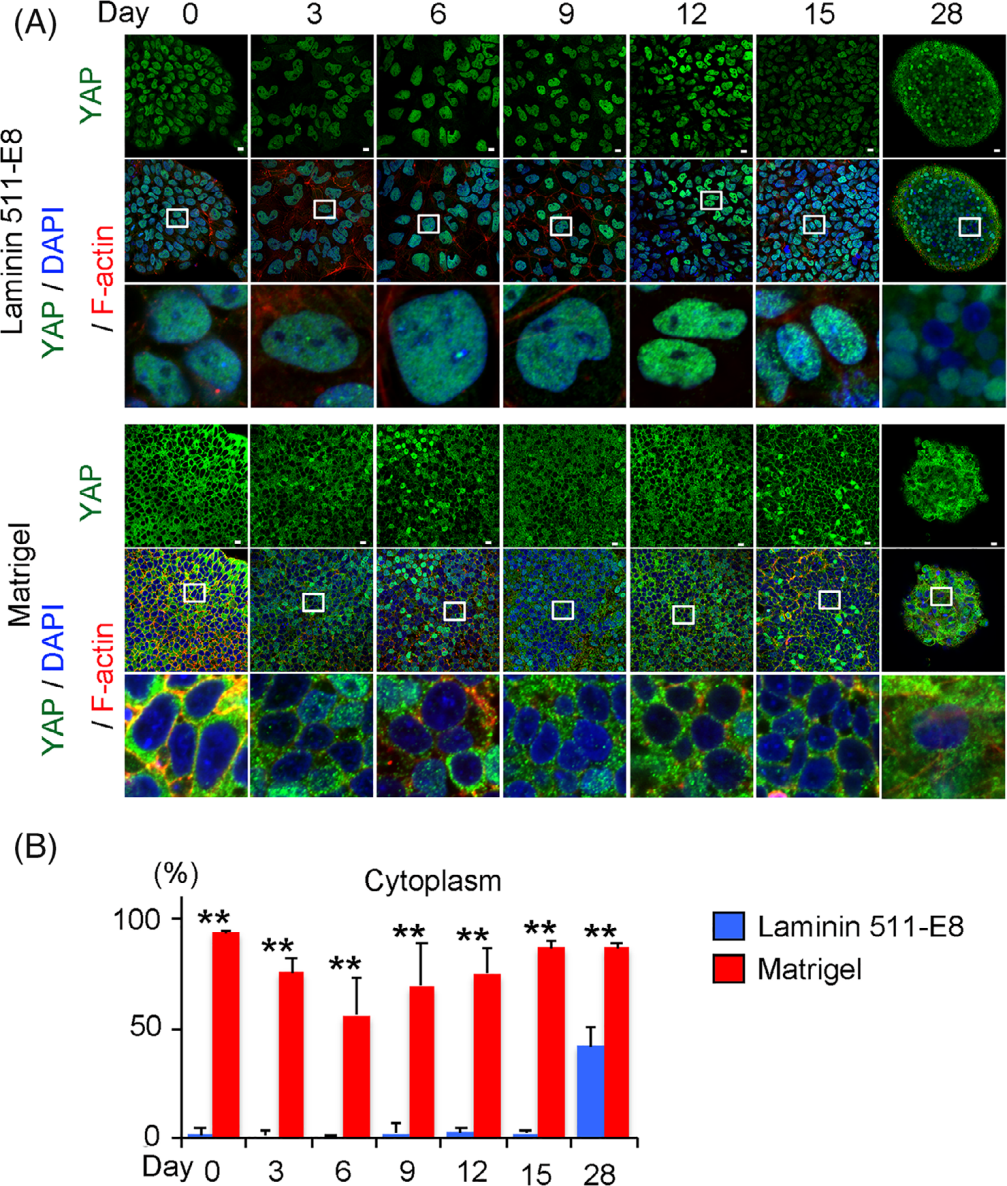
Comparison of YAP expression through chondrogenic differentiation between laminin and Matrigel conditions. A, YAP localization through chondrogenic differentiation in laminin‐coated dishes (*Top*) and Matrigel‐coated dishes (*Bottom*). The images were obtained with a confocal microscope. Anti‐YAP antibody (Green), 4',6‐diamidino‐2‐phenylindole (DAPI) (Blue) and anti F‐actin (Red). Scale bars = 10 μm. The data are representative of two independent experiments. Bottom rows are magnifications of the squares in the middle rows. B, Percentage of cells in which YAP is localized in the cytoplasm in a microscope field through chondrogenic differentiation (N = 6 microscope fields in one dish). Student's *t* test. **P < .01. YAP, yes‐associated protein

A confocal microscopy analysis of the iPSCs cultured either on laminin 511E8 or Matrigel for 7 days revealed that the average height of the cells was higher in the Matrigel group (Figure 1D). Because YAP was reported to correlate with the culture condition and cell morphology,^19^ we examined its activity. The active form of YAP resides in the nucleus and regulates gene transcription. Upon phosphorylation by the Hippo pathway, YAP translocates to the cytoplasm and is inactivated.^23^ Immunocytochemical analysis revealed that YAP was located in the nuclei of iPSCs cultured on laminin 511‐E8, but in the cytoplasm of iPSCs cultured on Matrigel (Figure 1E). Immunoblot analysis indicated decreased phosphorylation of YAP in the laminin 511‐E8 group (Figure 1F). The distinct patterns of YAP localization between the laminin 511‐E8 and Matrigel groups persisted even after changing from ESC medium to chondrogenic medium #1 for at least 15 days (Figure 2).

### 3.2 Association between bioreactors, YAP inactivation, and cartilage formation from hiPSCs

**FIGURE 3:**
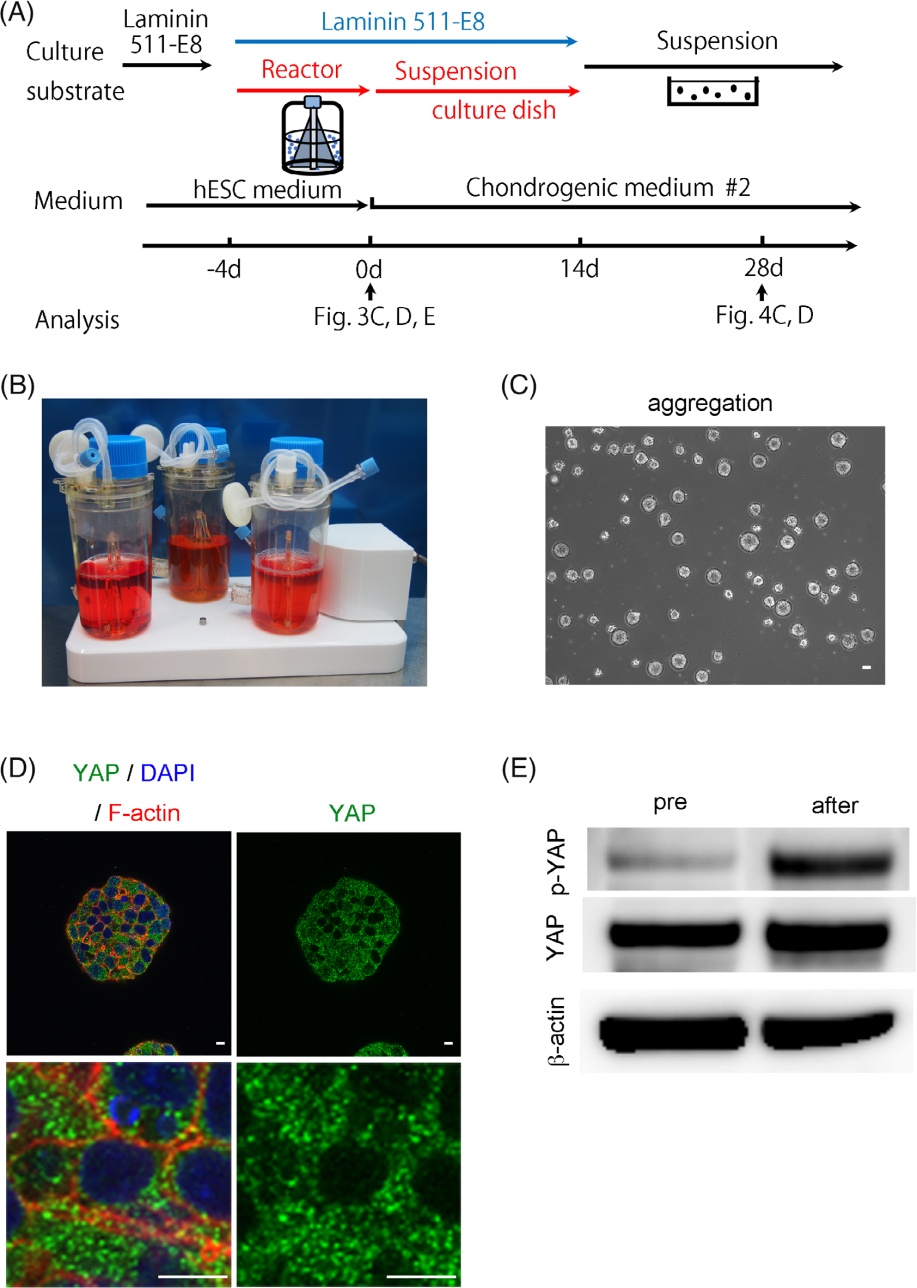
Use of a bioreactor in the chondrogenic differentiation of hiPSCs and analysis of YAP expression. A, Scheme for the chondrocyte differentiation using a bioreactor. B, Image of the bioreactors. C, Image of aggregates after 4 days culture in a bioreactor (day 0 in A). Scale bar = 10 μm. D, Immunostaining of an aggregate formed after 4 days culture of hiPSCs in a bioreactor for YAP in aggregates with confocal imaging. Anti‐YAP antibody (Green), 4',6‐diamidino‐2‐phenylindole (DAPI) (Blue), and anti F‐actin (Red). Scale bars = 10 μm. The data are representative of two independent experiments. E, Western blotting for phosphorylated YAP (p‐YAP), total YAP and β‐actin. The data are representative of three independent experiments. hiPSCs, human induced pluripotent stem cells; YAP, yes‐associated protein

**FIGURE 4:**
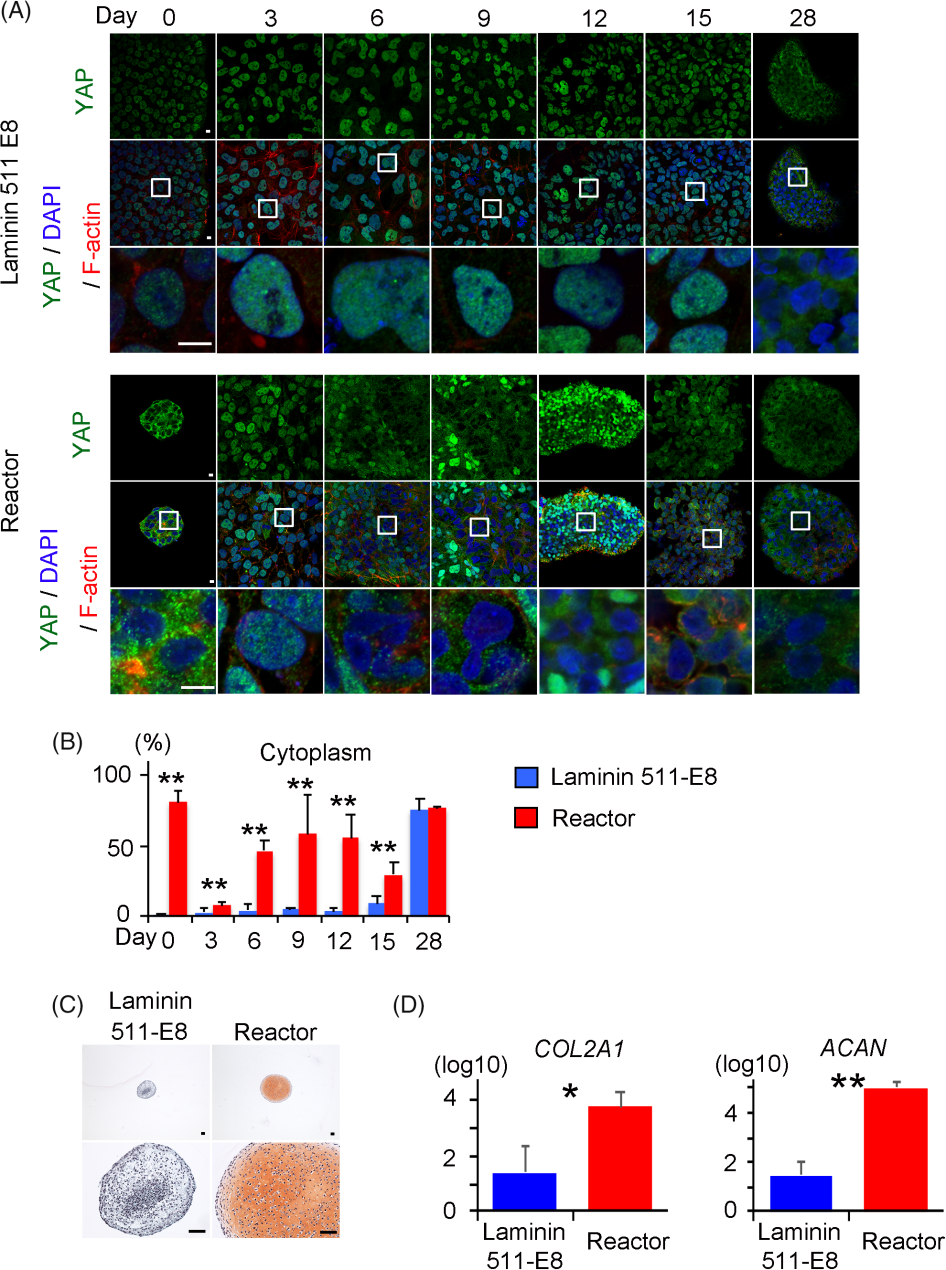
Comparison of YAP expression through chondrogenic differentiation and cartilage formation between the laminin and bioreactor conditions indicated in Figure 3A. A, YAP localization through chondrogenic differentiation in conditions using laminin‐coated dishes (*Top*) or the bioreactor (*Bottom*). The images were obtained with a confocal microscope. Anti‐YAP antibody (Green), 4',6‐diamidino‐2‐phenylindole (DAPI) (Blue), and anti F‐actin (Red). The data are representative of two independent experiments. Bottom rows are magnifications of the squares in the middle rows. B, Percentage of cells in which YAP is localized in the cytoplasm in a microscope field through chondrogenic differentiation (N = 6 microscope fields in one dish). Student's *t* test. **P < .01. C, Histology of particles on day 28 stained with Safranin‐O. Scale bars = 50 μm. One image is representative of three particles. D, Real‐time reverse transcription‐polymerase chain reaction (RT‐PCR) measurements of *COL2A1* and *AGGRECAN* (*ACAN*) expression in particles on day 28 (N = 3 independent experiments). Student's *t* test. *P < .05. **P < .01. YAP, yes‐associated protein

To confirm the association between YAP inactivation and cartilage formation, we used a bioreactor to pretreat hiPSCs before starting chondrogenic differentiation (Figure 3A,B), because it was reported that culturing NIH3T3 cells in suspension reduces the nuclear accumulation of YAP.^24^ Treatment of the hiPSCs in suspension culture in a bioreactor for 4 days resulted in the formation of cell aggregates (Figure 3C) and localization of YAP in the cytoplasm with increased phosphorylation (Figure 3D,E). We next put the aggregates into suspension culture dishes and cultured them in chondrogenic medium #2. We let the aggregates attach to the bottom of the dishes for 14 days and then scraped them off and transferred them into suspension culture. YAP transiently translocated into the nuclei 3 days after putting the aggregates into dishes but relocated to the cytoplasm after that (Figure 4A,B). The resultant particles were cartilaginous (Figure 4C,D), indicating an association between YAP inactivation and cartilage formation.

### 3.3 YAP activation prevents cartilage formation from hiPSCs cultured on laminin

**FIGURE 5:**
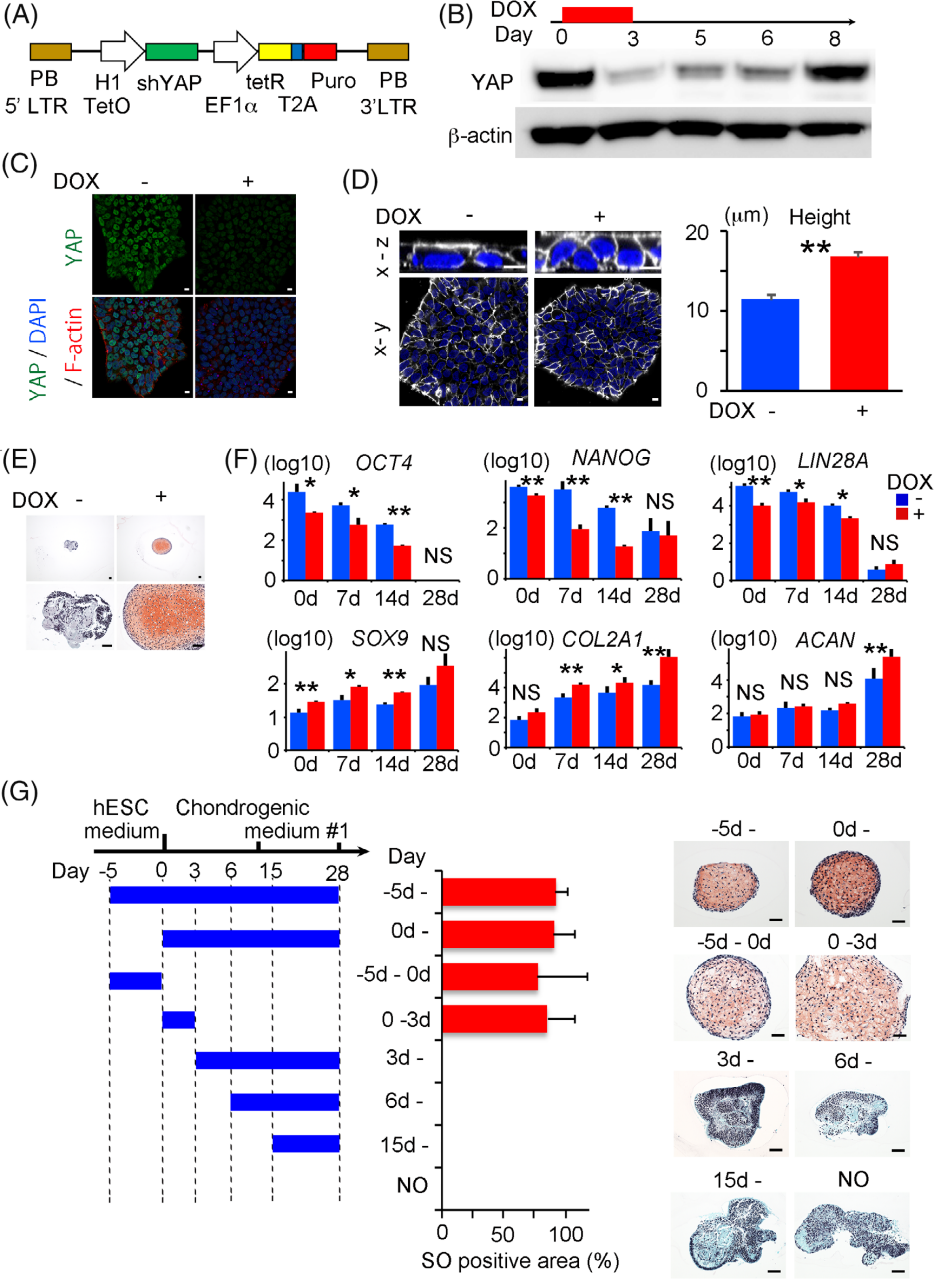
Downregulation of YAP in doxycycline‐inducible short hairpin (sh) YAP‐hiPSCs and chondrogenic differentiation using laminin 511‐E8. A, Structure of the doxycycline‐inducible YAP shRNA knockdown piggyBac vector, pPB‐H1TetOx1‐shYAP. B, shYAP‐hiPSCs on laminin 511‐E8 were cultured in the presence of doxycycline for 3 days and then in the absence of doxycycline for 5 days. Samples were recovered at the indicated time and subjected to Western blotting for total YAP and β‐actin. C,D, shYAP‐hiPSCs on laminin 511‐E8 were cultured in the presence or absence of doxycycline for 3 days (C) and 7 days (D) and subjected to analysis. C, Immunostaining for YAP in confocal imaging. Anti‐YAP antibody (Green), 4',6‐diamidino‐2‐phenylindole (DAPI) (Blue), and anti F‐actin (Red). The data are representative of two independent experiments. D, *Left*, Cell membrane staining using Cellbrite. Snapshots of the 3D reconstitution from the z‐series of the confocal images are shown. The upper and bottom images are the x‐z and x‐y views, respectively. Cell membrane (white) and Hoechst (blue). Scale bars = 10 μm. *Right*, Height of the hiPSCs colonies (N = 6 microscope fields in one dish). Student's *t* test. **P < .01. The data are representative of two independent experiments. E‐G, Analysis of particles generated by the chondrogenic differentiation of shYAP‐hiPSCs for 28 days using laminin 511‐E8. Doxycycline was present or absent from 5 days before the start of differentiation to day 28 (E, F) or for fixed periods (G). E, Histological sections of the particle at 28 days after the start of differentiation were stained with Safranin‐O. Scale bars = 50 μm. The data are representative of three independent experiments. F, Particles were periodically harvested at the indicated days after differentiation and subjected to Real‐time reverse transcription‐polymerase chain reaction (RT‐PCR) measurements of *POU5F1 (OCT4)*, *NANOG*, *LIN28A*, *SOX9*, *COL2A1*, and *AGGRECAN* (*ACAN*) expression (N = 3 independent experiments). Student's *t* test. *P < .05. **P < .01. G, Periods for which doxycycline was added are indicated by blue bars. Histological sections stained with Safranin‐O (SO). Scale bars = 50 μm. The percentage of the SO‐positive area to the total area of the particle was measured (n = 3 independent experiments). hiPSCs, human induced pluripotent stem cells; shRNA, short hairpin RNA; YAP, yes‐associated protein

To examine whether YAP activation prevents cartilage formation, we knocked down YAP during the chondrogenic differentiation of hiPSCs cultured on laminin 511‐E8. We created the shYAP‐hiPSC line bearing a doxycycline‐inducible YAP knockdown transgene (Figure 5A) by using the PiggyBac transposon vector system. The expression of YAP in shYAP‐hiPSCs was decreased by 3 days after the addition of doxycycline and recovered by 5 days after the withdrawal of doxycycline (Figure 5B). YAP localized in the nuclei of hiPSCs cultured on laminin 511‐E8 in the absence of doxycycline, but not in the presence of doxycycline (Figure 5C). Furthermore, the addition of doxycycline increased the average height of shYAP‐hiPSCs cultured on laminin 511‐E8 (Figure 5D). We then subjected the cells to chondrogenic differentiation in the absence and presence of doxycycline. The resultant particles after chondrogenic differentiation for 28 days were not cartilaginous in the absence of doxycycline, but were cartilaginous in the presence, as indicated by safranin O staining (Figure 5E) and the expressions of *COL2A1* and *ACAN* (Figure 5F). Interestingly, treatment with doxycycline for 5 days decreased the expression levels of pluripotent markers and increased the expression level of *SOX9* in shYAP‐hiPSCs before the chondrogenic differentiation (Figure 5F, day 0). These results suggest that YAP activation prevents cartilage formation in the chondrogenic differentiation of hiPSCs cultured on laminin 511‐E8.

**FIGURE 6:**
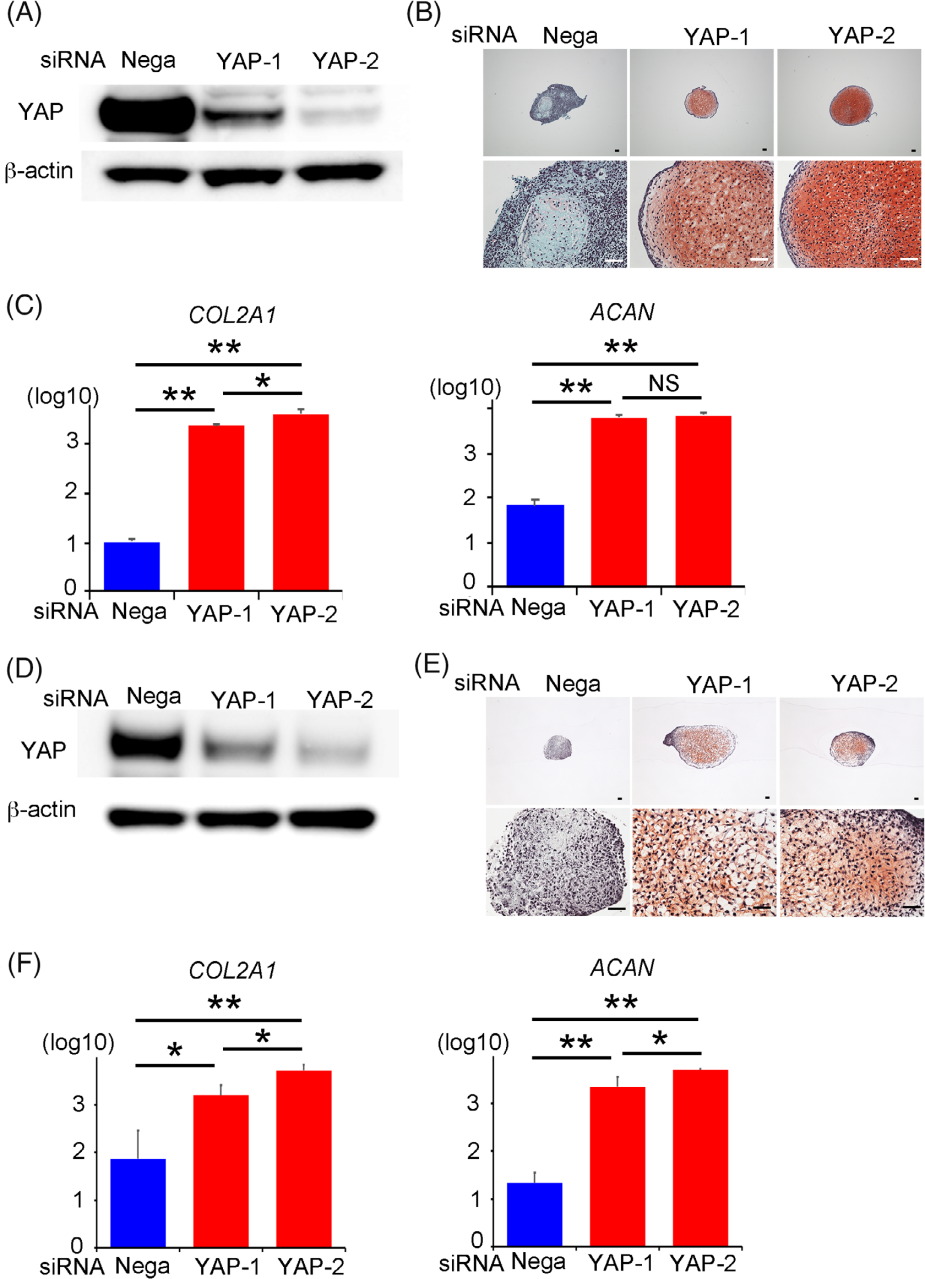
Downregulation of YAP in QHJI hiPSCs (A‐C) and 604B1 hiPSCs (D‐F) by siRNA and chondrogenic differentiation. A,D, Western blotting for total YAP and β‐actin 3 days after the transfection of hiPSCs with YAP siRNA. The data are representative of three independent experiments. B,C,E,F, hiPSCs that were transfected with YAP siRNA were subjected to chondrogenic differentiation using laminin 511‐E8. The particles formed on day 28 were analyzed. B,E, Histological sections stained with Safranin‐O. Scale bars = 50 μm. One image is representative of three particles. C,F, Real‐time reverse transcription‐polymerase chain reaction (RT‐PCR) measurements of *COL2A1* and *AGGRECAN* (*ACAN*) expression (N = 3 independent experiments). Student's *t* test. *P < .05; **P < .01. hiPSCs, human induced pluripotent stem cells; YAP, yes‐associated protein

To narrow down the necessary period in which YAP is knocked down for cartilage formation, we treated the shYAP‐hiPSCs with doxycycline for fixed periods during chondrogenic differentiation. Treatment with doxycycline either for 5 days before the start of chondrogenic differentiation or 3 days after resulted in cartilage formation (Figure 5G), suggesting that the inactivation of YAP around the start of chondrogenic differentiation is important. This finding was confirmed by the result that cartilage was formed from hiPSCs cultured on laminin 511‐E8 when YAP was inactivated from the start of chondrogenic differentiation by siRNAs that knock down the target mRNAs only transiently (Figure 6A‐C). To generalize these findings across different iPSC lines, we tested another iPSC line, 604B1. Knocking down YAP by siRNAs improved the generation efficiency of cartilaginous particles in this line too (Figure 6D‐F).

**FIGURE 7:**
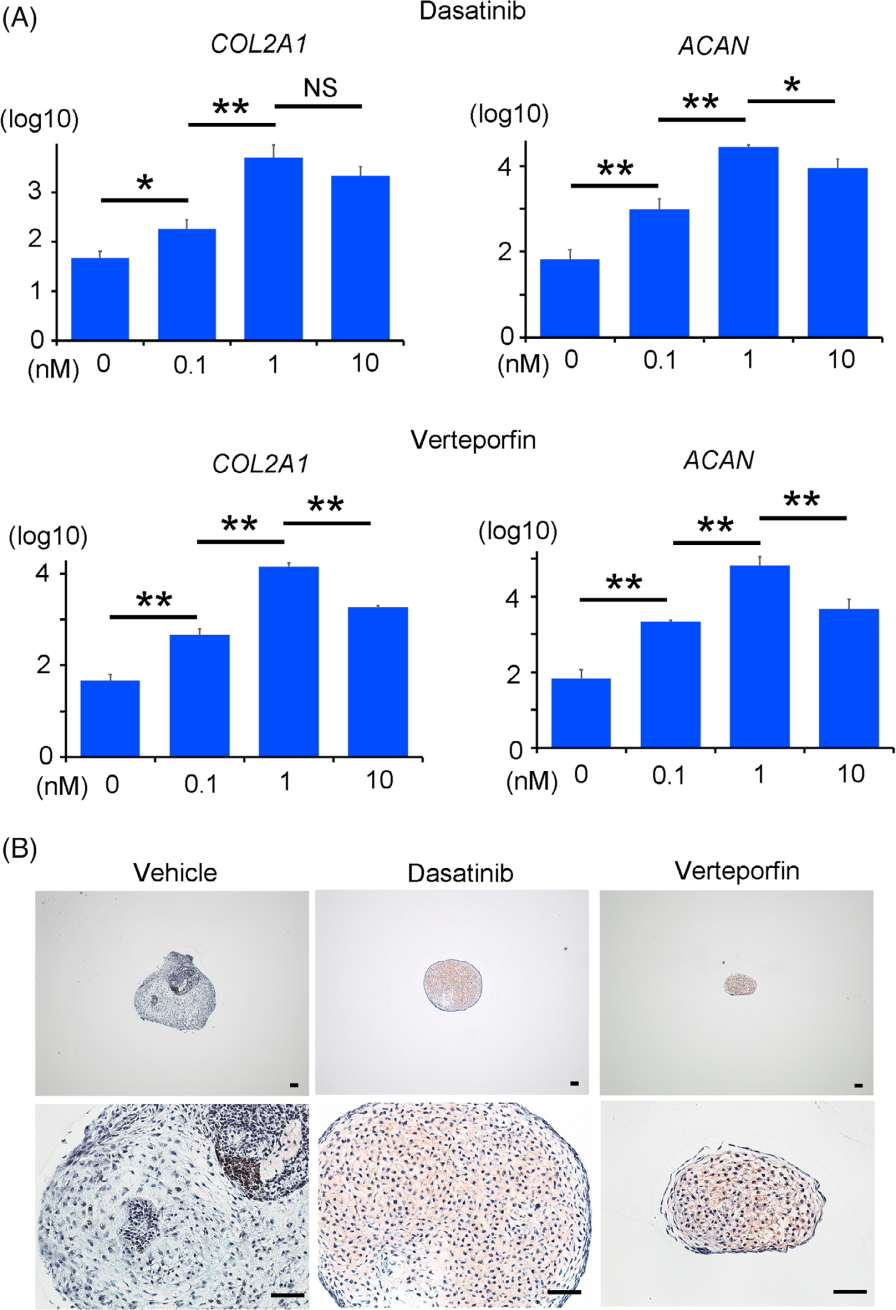
Chondrogenic differentiation of hiPSCs in the presence of a YAP inhibitor. hiPSCs were subjected to chondrogenic differentiation using laminin 511‐E8 in the absence or presence of Dasatinib or Verteporfin at final concentrations of 0.1, 1, and 10 nM (A) and 1 nM (B). The particles formed on day 28 were analyzed. A, Real‐time reverse transcription‐polymerase chain reaction (RT‐PCR) measurements of *COL2A1* and *AGGRECAN* (*ACAN*) expression (N = 3 independent experiments). Student's *t* test. *P < .05; **P < .01. B, Histological sections stained with Safranin‐O. Scale bars = 50 μm. One image is representative of three particles. hiPSCs, human induced pluripotent stem cells; YAP, yes‐associated protein

Finally, we examined whether the inhibition of YAP with small compounds produces cartilage. The addition of either Dasatinib or Verteporfin improved the expression levels of chondrocytic markers with the better improvement at 1 nM than at 0.1 and 10 nM in the particles formed from hiPSCs cultured on laminin 511‐E8 (Figure 7A). The particles formed from iPSCs in the presence of 1 nM Dasatinib or Verteporfin were stained with safranin O positively but weakly (Figure 7B), indicating the degree of the improvement was limited.

## 4 DISCUSSION

In this study, we examined the effects of culture substrates such as laminin 511‐E8 and Matrigel on the formation of cartilage of chondrogenically differentiated hiPSCs. Cartilage formation was achieved with Matrigel as the substrate, but Matrigel contains many unknown components, prohibiting its use for clinical settings including hiPSC‐derived cartilage therapies.^16^ On the other hand, cartilage formation was not achieved when using laminin 511‐E8 as the substrate, a result associated with the activation and intranuclear localization of YAP. Indeed, the concomitant knockdown of YAP or the addition of a YAP inhibitor resulted in complete and partial cartilage formation, respectively. The addition of chemical inhibitors is more acceptable than the transfection of siRNA vectors in clinical settings from the viewpoint of regulation. The observed incomplete cartilage formation by the addition of YAP inhibitors in the present study is probably due to off‐target effects of the inhibitors. Further analysis is needed to identify these off‐target effects and to compensate for them by modulating the composition of the chondrogenic medium in order to obtain cartilage that is clinically acceptable. The use of bioreactors, for example, is clinically acceptable, and our results suggest that the combination of bioreactors and laminin can replace Matrigel for the purpose of producing hiPSC‐derived cartilage.

We also found that iPSCs were flat (low cell height) and YAP was active when cultured on laminin 511‐E8, but they were round and YAP was inactive when cultured on Matrigel or in a bioreactor. It was reported that cells spread out and YAP is active when cultured on a stiff substrate, but round and YAP is inactive when cultured on a soft substrate.^19^ Thus, our results suggest that difference in the mechanical stiffness of the substrates is a factor that regulates cell shape and YAP activity. Other factors may include various substances in Matrigel and fluid flow shear stress in bioreactors.

Although we found that YAP inactivation in the early phase of the differentiation process is responsible for cartilage formation, the precise mechanism for how YAP inactivation stimulates chondrogenic differentiation in hiPSCs remains elusive. YAP is involved in cell proliferation and apoptosis,^13^, ^25^, ^26^ and the activation of YAP/TAZ contributes to the survival and cellular identity of human ESCs.^14^, ^15^, ^27^ Consistently, our results indicated that YAP knockdown decreased the expression levels of pluripotent markers and increased the expression level of *SOX9* in hiPSCs, suggesting that YAP knockdown compromises the pluripotency of hiPSCs and induces their differentiation. Based on previous reports and our results, we speculate that the inhibition of YAP disrupts the transcriptional network maintaining the undifferentiated state of hiPSCs, making the cells vulnerable to differentiation including chondrogenic differentiation.

In summary, we clarified the effects of culture substrates on cartilage formation from iPSCs. Different substrates were associated with different levels of YAP activation, which we found play a crucial role in cartilage formation. This connection between culture conditions and the activities of intracellular molecules on cartilage formation contributes to our understanding of the molecular mechanisms that promote hiPSC chondrogenic differentiation and will assist in establishing efficient protocols for the preparation of implantable cartilage into patients.

## CONFLICT OF INTEREST

The authors declared no potential conflicts of interest.

## AUTHOR CONTRIBUTIONS

A.Y.: conception and design, financial support, acquisition of data, data analysis and interpretation, drafting manuscript; N.T.: conception and design, financial support, data analysis and interpretation, drafting manuscript; S.K.: acquisition of data; H.Y., J.T.: construction of a knockdown vector.

## ETHICS STATEMENT

All methods were carried out in accordance with relevant guidelines and regulations. Experiments using recombinant DNA were approved by the Recombinant DNA Experiments Safety Committee of Kyoto University. Research involving human subjects was approved by the Ethics Committee of Kyoto University. Written informed consent was obtained from each donor.

## Data Availability

The data that support the findings of this study are available on request from the corresponding author.
